# Point CNN:3D Face Recognition with Local Feature Descriptor and Feature Enhancement Mechanism

**DOI:** 10.3390/s23187715

**Published:** 2023-09-06

**Authors:** Qi Wang, Hang Lei, Weizhong Qian

**Affiliations:** School of Information and Software Engineering, University of Electronic Science and Technology of China, Chengdu 610054, China

**Keywords:** face recognition, convolutional neural network, point clouds, local feature descriptor

## Abstract

Three-dimensional face recognition is an important part of the field of computer vision. Point clouds are widely used in the field of 3D vision due to the simple mathematical expression. However, the disorder of the points makes it difficult for them to have ordered indexes in convolutional neural networks. In addition, the point clouds lack detailed textures, which makes the facial features easily affected by expression or head pose changes. To solve the above problems, this paper constructs a new face recognition network, which mainly consists of two parts. The first part is a novel operator based on a local feature descriptor to realize the fine-grained features extraction and the permutation invariance of point clouds. The second part is a feature enhancement mechanism to enhance the discrimination of facial features. In order to verify the performance of our method, we conducted experiments on three public datasets: CASIA-3D, Bosphorus, and Lock3Dface. The results show that the accuracy of our method is improved by 0.7%, 0.4%, and 0.8% compared with the latest methods on these three datasets, respectively.

## 1. Introduction

Face recognition, as an important part of the field of computer vision, is widely used in daily life. However, most related studies are based on common RGB images and it is difficult for common digital cameras to obtain effective RGB images under the condition of large illumination changes [1]. The devices of point clouds often do not rely on visible light, such as lidar and Kinect (based on infrared), which makes this kind of data stable to illumination changes and can be applied to some special scenes. The mathematical expression of the point clouds is simple (a group of points in 3D space). However, the disorder of the point clouds makes it difficult for them to have an ordered index such as ordinary 2D images, so it is difficult to use deep learning networks for feature extraction [2]. Deep learning is widely used in various research fields due to its powerful perception. Refs. [3,4] applied deep learning to real engineering technology and achieved outstanding performances. As the pioneers Qi et al. [5] used the symmetric function to construct PointNet that solved the disorder of point clouds in deep learning, many networks based on PointNet have been proposed, such as PointNet++ [6], ppfnet [7], pointcnn [8], etc. Subsequently, point clouds are also widely used in face analysis tasks, such as face detection, pose estimation, face recognition and verification, etc. Particularly in the field of face recognition, a large number of methods have been proposed. However, due to the lack of detailed textures in the point clouds, the fine-grained expression of facial features is still the focus of research in this field. Relying on powerful perception capabilities, convolutional neural networks (CNNs) have made breakthroughs in the field of 2D images. In order to make the point clouds effectively utilize the perceptual power of CNNs, Li et al. [8] constructed a convolution operator, which realizes the permutation invariance of the disordered points through a permutation matrix. Based on [7,8], we utilize the convolution operator with a local feature descriptor to construct a new operator, ψ−conv, to extract fine-grained features of point faces. Furthermore, we propose a novel feature enhancement mechanism to further enhance the discrimination of facial features and introduce a triplet loss function based on the feature enhancement mechanism for efficient 3D face recognition.

In order to verify the effectiveness of our method, we conduct experiments on three public datasets: CASIA-3D, Bosphorus, and Lock3Dface. 

The main novelty and contribution of this paper are summarized as follows:

1. We construct a new operator based on local feature descriptors to achieve fine-grained feature extraction from disordered point clouds;
2. A new feature enhancement mechanism is introduced, which effectively improves the accuracy of the point face recognition;
3. The experimental results on public datasets prove that the accuracy of our proposed method outperforms current advanced algorithms. Additionally, our method can better deal with the interference of facial expressions, partial occlusions, and head pose changes.

## 2. Related Works

In this section, we briefly review some typical and relevant works in the field of 2D face recognition and 3D face recognition. 

### 2.1. Two-Dimensional Face Recognition

In recent years, the most widely used face recognition methods have mainly been proposed on 2D RGB images. Schroff et al. [9] used a convolutional neural network to extract features and introduced the triplet loss function to build the famous FaceNet for RGB face recognition, which outperforms humans in accuracy. In order to deal with the occlusion and illumination variations, Yang et al. [10] presented a 2D image matrix-based error model (NMR) for face representation and classification. Focusing on the illumination change challenge, Guo et al. [11] proposed a deep network model that takes both visible light images and near-infrared images into account to perform face recognition. Unlike conventional feature descriptors, Lu et al. [12] proposed a new joint feature learning (JFL) approach to automatically learn feature representation from raw pixels for face recognition. Deng et al. [13] proposed an additive angular margin loss to obtain highly discriminative features for face recognition. Aiming at inferring genuine emotions from micro-expression recognition, Zong et al. [14] designed a hierarchical spatial division scheme for spatiotemporal descriptor extraction. Wenhui et al. [15] studied the combination of 2D discriminant analysis and 1D discriminant analysis and proposed a stable framework MMC + LDA for face recognition. Zhang et al. [16] proposed a high-order local pattern descriptor (LDP) for face recognition, which achieves good performance under various conditions.

### 2.2. Three-Dimensional Face Recognition

With the development of 3D sensors, more and more methods have been proposed for 3D face analysis. Zhang et al. [17] proposed a general approach to deal with the 3D face recognition problem by making use of multiple key point descriptors (MKD) and the sparse representation-based classification (SRC). Chouchane et al. [18] presented an automatic face recognition system in the presence of illumination, expressions, and pose variations based on 2D and 3D information. In [19], Szegedy et al. explored ways to scale up CNNs that aimed at utilizing the added computation for computer vision. In order to optimize deeper neural networks for image recognition, He et al. [20] presented a residual learning framework to ease the training of networks. Based on local derivative pattern (LDP), Soltanpour et al. [21] proposed a descriptor for 3D face recognition. Focusing on the intrinsic invariance to pose and illumination changes, Mu et al. [22] designed a lightweight yet powerful CNN with low-quality data to achieve an efficient and accurate deep learning solution. Dutta et al. [23] constructed a sparse principal component analysis network (SpPCANet) to extract 3D face features for recognition. 

In the field of 3D vision, as the PointNet [5] solves the disorder of point clouds in deep learning, these kinds of data are widely used with their simple mathematical expression; more algorithms are proposed for 3D face recognition. Bhople et al. [24] combined PointNet and Siamese network for similarity learning of point faces and have achieved encouraging performances in the field of face recognition. Atik et al. [25] mapped point clouds to feature maps and used 2D methods to solve 3D face recognition. In order to enhance the robustness of the 3D point cloud face recognition system for multiple expressions and multiple poses, Gao et al. [26] used point clouds as input and constructed a deep learning feature extraction network, ResPoint. Yu et al. [27] modified PointNet and supplemented a few data-guided learning frameworks based on a Gaussian process morphable model for 3D face recognition. Cao et al. [28] utilized PointNet++ and RoPS local descriptors to extract local features of a 3D face. In order to deal with the lack of large-scale 3D facial data, Zhang et al. [29] established a statistical 3D morphable model-based 3D face synthesizing strategy to generate large-scale unreal facial scans to train the proposed network. Yu et al. [30] proposed a meta learning-based adversarial training (MLAT) algorithm for deep 3D face recognition on point clouds, which consists of two alternate modules: adversarial sample generating for 3D face data augmentation and meta learning-based deep network training. Jiang et al. [31] used two weight-shared encoders and a feature similarity loss to guide the encoders to obtain discriminative face representations and have achieved good performance on different datasets. Apart from face recognition, point clouds are also used for other 3D face analysis tasks such as face verification and head pose estimation [1,2,32].

## 3. Methods

The convolutional neural network (CNN) is highly invariant to image translation, scaling, and tilting through multi-layer feature extraction and regional weight sharing [8]. However, due to the disorder of the point clouds, a CNN cannot directly perform feature extraction on them. In this section, firstly, we introduce a local feature descriptor for fine-grained feature representation and then introduce the ψ−conv for the convolution operation of the point clouds. Thirdly, depending on the ψ−conv, we construct a new convolutional neural network for facial feature extraction. Fourthly, a new feature enhancement mechanism is proposed to enhance the discrimination of facial features. Finally, based on the feature enhancement mechanism, we adopt a triplet loss function for training and construct an efficient face recognition network.

### 3.1. Local Feature Descriptor

In this part, inspired by [7], in order to obtain the fine-grained representation of features, we use a hand-crafted descriptor to describe the local geometric features of the point clouds.

For a points pair $\left(p_{i},p_{j}\right)$, the geometric relationship between two points is represented by a four-dimensional descriptor:

(1) $$\psi_{ij}=\left(||d{||}_{2},∠\left(n_{i},d\right),∠\left(n_{j},d\right),∠\left(n_{i},n_{j}\right)\right)$$

where $||d_{2}||$ represents the Euclidean distance between two points. The $n_{i}$ and $n_{j}$ are normal vectors of $p_{i}$ and $p_{j}$, respectively. The ∠ is the angle between two vectors:

(2) $$∠\left(v_{i},v_{j}\right)=\tan^{-1}(\frac{||v_{i}\times v_{j}||}{v_{i}\cdot v_{j}})$$

where $∠\left(v_{i},v_{j}\right)\in \left[0,\pi \right)$, the “×” represents the cross-product and the “⋅” represents the dot-product. As described above, the $\psi_{ij}$ describes in detail the geometric relationship between two points through normal vectors and angles.

**Figure 1:**
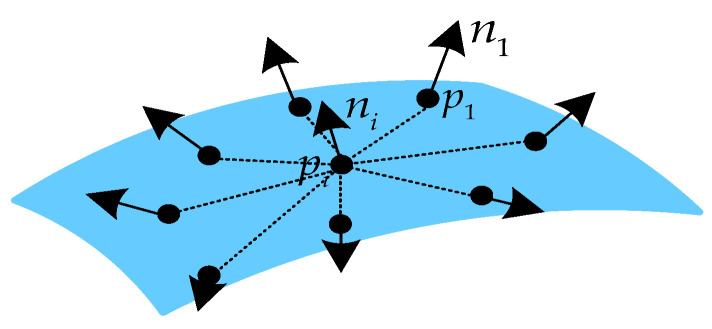
Example of the local feature descriptor with the center point $p_{i}$.

For a local region, $\left\{p_{1},p_{2},p_{3},\cdot \cdot \cdot ,p_{n}\right\}$, we choose a center point $p_{i}$, which has a total of *n* pairs of points (including $\left(p_{i},p_{i}\right)$); the geometric feature of this local region is expressed as follows:

(3) $$F_{i}=\left[p_{1},n_{1},p_{2},n_{2},\ldots ,p_{j},n_{j},\psi_{i1},\psi_{i2}\ldots ,\psi_{ij}\right]$$

where $p_{j}$ is the point in the local region and $n_{j}$ is the normal vector of point $p_{j}$. The $\psi_{ij}$ is the four-dimensional descriptor between $p_{j}$ and center point $p_{i}$. As shown in Figure 1, $F_{i}$ uses all points pairs with the center point $p_{i}$ to describe the spatial geometric characteristics of the local region.

### 3.2. ψ−Conv Operator

**Figure 2:**
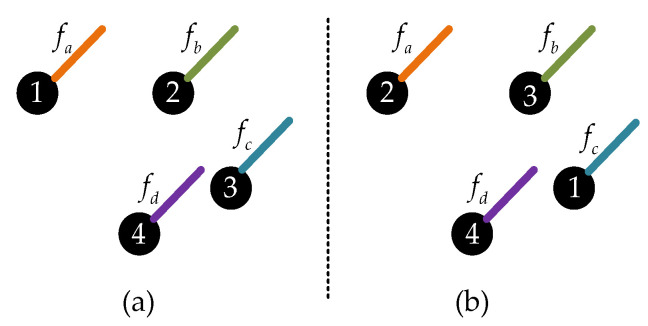
Example of the disorder of the point clouds, where (**a**,**b**) represent point clouds with different index orders under the same distribution.

As mentioned above, because of the disorder of the point clouds, they cannot directly use the convolution operation. To deal with the problem, Li et al. [8] trained a permutation matrix through a multi-layer perceptron (MLP) to realize the permutation invariance of the point clouds. As shown in Figure 2, the points in Figure 2a,b have the same distribution but the orders are different.

In Figure 2, $f_{a}$, $f_{b}$, $f_{c}$, $f_{d}$ represent the features of the corresponding points and the number represents the order of each point. We use a same convolution kernel $K={\left[k_{\alpha },k_{\beta },k_{\gamma },k_{\delta }\right]}^{T}$ to operate on the above two point clouds:

(4) $$f_{\left(a\right)}=Conv(K,\left[f_{a},f_{b},f_{c},f_{d}{]}^{T}\right)$$

(5) $$f_{\left(b\right)}=Conv(K,\left[f_{c},f_{a},f_{b},f_{d}{]}^{T}\right)$$

(6) $$f_{\left(a\right)}\neq f_{\left(b\right)}$$

**Figure 3:**
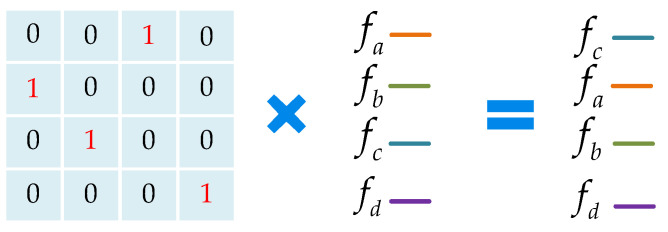
The permutation matrix to adjust the order of the points.

As shown above, the two sets of point clouds have the same distribution, but the convolution results are different. As shown in Figure 3, in order to make the convolution result only related to the distribution but not to the order, we use a permutation matrix to adjust the order of the points.

Based on the local feature descriptor and the permutation matrix, we construct a new operator ψ−conv, which achieves permutation invariance and fine-grained feature extraction of a local region of the point clouds. The algorithm of the ψ−conv operator is as follows in Algorithm 1 below:
**Algorithm 1** ψ−conv operatorInput: *P*, *p*, *K*
Output: $F_{p}$

$1:P^{*}\leftarrow P-p$Local coordinate transformation.$2:\left\{\psi_{1},\psi_{2},\cdot \cdot \cdot ,\psi_{n}\right\}\leftarrow \left(P^{*},\;p\right)$Encode point pairs with the descriptor $\psi_{ij}$.$3:F_{l}\leftarrow \left[\psi ,P^{*}\right]$Local feature descriptor.$4:F_{\beta }\leftarrow PointNet\left(F_{l}\right)$PointNet to extract local geometric features.$5:F_{\alpha }\leftarrow MLP_{\alpha }\;\left(P^{*}\right)$$MLP_{\alpha }$ performs point-by-point feature extraction.$6:F_{*}\leftarrow \left[F_{\beta },F_{\alpha }\right]$Concatenate $F_{\beta },F_{\alpha }$.$7:\chi \leftarrow MLP_{\chi }\;\left(P^{*}\right)$Obtain weight matrix χ through $MLP_{\chi }$.$8:F_{\chi }\leftarrow \chi \times F_{*}$Achieving feature permutation invariance.$9:F_{P}\leftarrow Conv\left(K,F_{\chi }\right)$Feature extraction using the convolution kernel *K*.

The input of ψ−conv is the set of feature points in the local region $P=\left\{p_{1},p_{2},p_{3},\ldots ,p_{k}\right\}$ and *p* is the center of *P* (we take *p* as the center and use the k-nearest neighbors algorithm (KNN) to sample the nearest *k* points, $p\in {\mathbb{R}}^{C1}$). *K* represents the convolution kernel and the size of *K is k* (the size of the convolution kernel is the same as the number of points in the local region).

In the first step, the spatial coordinates of $P=\left\{p_{1},p_{2},p_{3},\ldots ,p_{k}\right\}$ are transformed into relative coordinates based on the center point *p* (relative coordinates make local points translation invariant).

The second step is to encode the points pairs in the local region according to Formula (1).

In the third step, according to Formula (3), the local feature descriptor is used to encode the local geometric feature.

**Figure 4:**
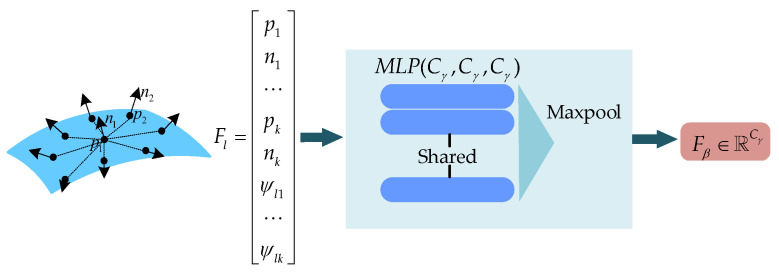
The PointNet for local feature extraction.

The fourth step, the PointNet is used to extract local geometric features. The structure of the PointNet is shown in Figure 4.

The PointNet consists of an MLP and a max pooling layer. The MLP has three layers and the number of nodes in each layer is the same, all of which are $C_{\gamma }$. After feature extraction, we obtain a local feature $F_{\beta }\in {\mathbb{R}}^{C_{\gamma }}$.

**Figure 5:**
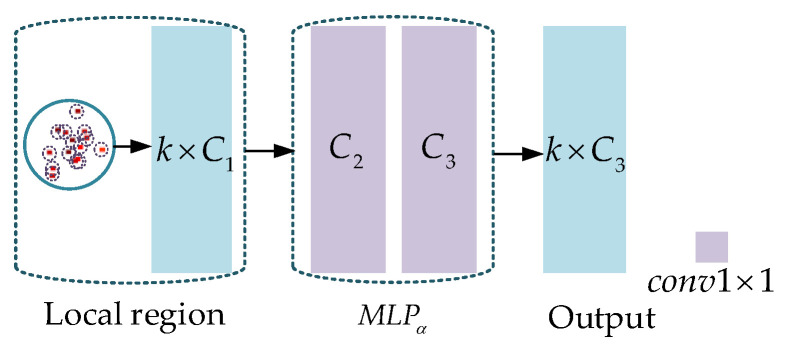
The structure of $MLP_{\alpha }$.

In the fifth step, we use the $MLP_{\alpha }P^{*}$ to improve the feature dimension of each point. The structure of the $MLP_{\alpha }$ is shown as Figure 5.

In Figure 5, *k* is the point number of the local region and $C_{1}$ is the feature dimension of the points. $MLP_{\alpha }$ has two convolutional layers. Due to the disorder of the points, only the 1×1 convolution kernel can be used to increase the dimension of the points (point-by-point). The number of channels of the two convolutional layers is $C_{2}$ and $C_{3}$, respectively ($C_{3}$ is the output dimension, $C_{2}=\left(C_{1}+C_{3}\right)/2$).

In the sixth step, the high-dimensional feature $F_{\alpha }$ of each point obtained in the fourth step is concatenated with the local geometric feature $F_{\beta }$ obtained in the fourth step (each point has the same $F_{\beta }$).

**Figure 6:**
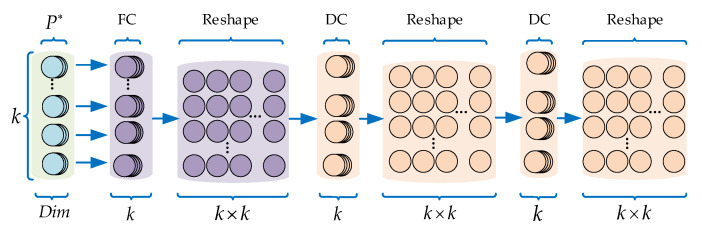
The structure of $MLP_{\chi }$.

In the seventh step, according to [8], we use $MLP_{\chi }$ to train a permutation matrix (as shown in Figure 3, which is only related to the distribution of points; *k* is the number of points in the local region) that redistributes the weight of each point to eliminate the influence of different orders. The structure of the $MLP_{\chi }$ is shown in Figure 6.

In Figure 6, a fully connected layer (FC) map *k* points (${\mathbb{R}}^{Dim}$) to k*k:$FC\left(Dim*k\to k*k\right)$ and reshapes it into a k×k matrix. Then, we adopt two layers of depth-wise convolution (DC, different from normal convolutional layer, the kernel of depth-wise convolution is responsible for one channel and the feature map has the same number of channels as the input layer) and reshape the feature maps; a k×k permutation matrix χ can be obtained:

(7) $$DC\left(k\times k\to k*k\right)\to reshape\left(k*k\to k\times k\right)\to \;DC\left(k\times k\to k*k\right)\to reshape\left(k*k\to k\times k\right)\to \chi$$

**Figure 7:**
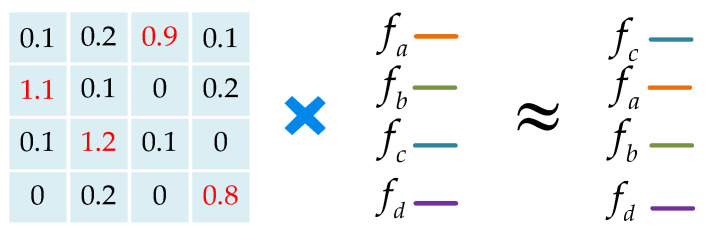
The weight matrix for permutation invariance.

Ideally, the permutation matrix is a binary matrix, as shown in Figure 3, but the obtained matrix by $MLP_{\chi }$ is a weight matrix, as shown in Figure 7. The weight matrix can approximate the permutation invariance of the local region.

The eighth step, $F_{\chi }\leftarrow \chi \times F_{*}$, where χ is the weight matrix obtained in the seventh step, $F_{*}$ is the concatenated feature of each point in the sixth step, and the “×” represents matrix multiplication. During this step, as shown in Figure 7, the point clouds achieve permutation invariance through the weight matrix χ and obtain the weighted features $F_{\chi }$ of the local region.

The ninth step, we can directly perform convolution operation on $F_{\chi }$ to obtain $F_{P}$ (feature map of this local region).

The above steps can be represented as follows:

(8) $$F_{p}=\psi -Conv\left(K,p,P\right)\;=Conv(K,MLP_{\chi }\left(P-p\right)\times \left[MLP_{\alpha }\left(P-p\right),PointNet\left(P-p\right)\right]$$

where *K*, *p*, and *P* represent the input of ψ−conv, $Conv\left(\cdot ,\cdot \right)$ is the convolution operation, and *PointNet*, $MLP_{\alpha }$, and $MLP_{\chi }$ are shown in Figure 4, Figure 5 andFigure 6, respectively.

### 3.3. CNN for Feature Extraction

**Figure 8:**
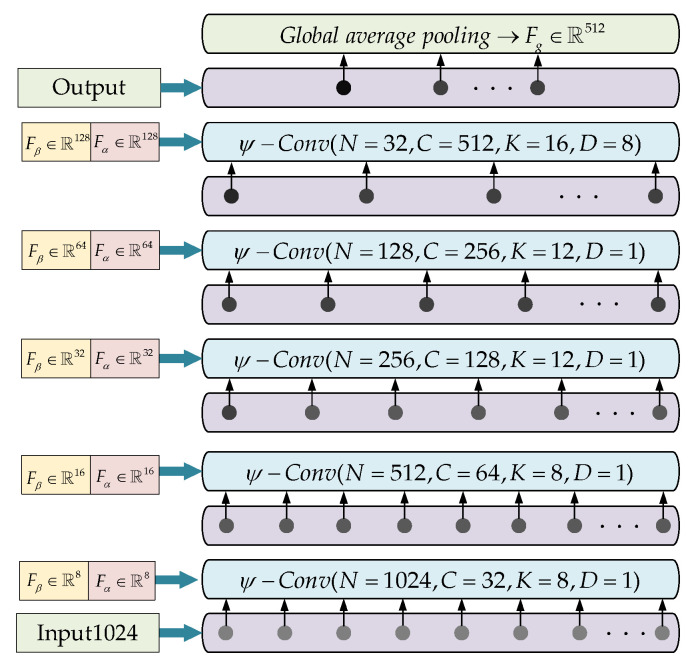
The convolutional neural network for facial feature extraction.

In Section 3.2, we use the local feature descriptor to describe the local fine-grained feature of a local region of the point clouds and adopt ψ−conv to weight the disordered point clouds to achieve the permutation invariance. In this section, based on ψ−conv, we construct a convolutional neural network (CNN) for facial feature extraction. The structure of our network is shown in Figure 8.

The network consists of 5 convolutional layers; the parameters of each layer are shown in Figure 8, where *K* is the number of points in a local region in this layer, *C* is the output feature dimension, *N* is the number of feature points in the next layer, and *D* is the dilation rate, which determines the receptive filed of the convolutional layer: $\left(K\times D\right)/N_{p}$ ($N_{p}$ is the number of feature points in the previous layer). For each layer, we also list the dimension of $F_{\beta }$, $F_{\alpha }$, which presents the size of the PointNet in Figure 4 and $MLP_{\alpha }$ in Figure 5.

Take the first layer as an example. The input point cloud has 1024 points (in our method, according to [32] we use farthest point sampling (FPS) algorithm sample 1024 points for each face). We use k-nearest neighbors algorithm (KNN) to sample 8 nearest points for each point (each local region has 8 points), then we adopt ψ−conv to extract the $F_{p}$ (convolution result, feature map) of each local region, where $F_{\beta }\in {\mathbb{R}}^{8}$, $F_{\alpha }\in {\mathbb{R}}^{8}$, which represent the $C_{\gamma }$ in the PointNet of this layer is 8 (as shown in Figure 4) and $C_{3}$ in the $MLP_{\alpha }$ of this layer is 8 (as shown in Figure 5). After the ψ−conv operation, each local region becomes a feature map $F_{p}\in {\mathbb{R}}^{32}$ and is regarded as a new point in ${\mathbb{R}}^{32}$ for the next convolution layer.

After 5 convolution layers, the number of feature points changes as follows: 1024→1024→512→256→128→32. The feature dimension changes as follows: 3→32→64→128→256→512. In the last convolutional layer, the receptive filed $\left(K\times D\right)/N_{p}=1$, which means the last 32 feature points “see” the whole region of the previous layer. Then, we use a global average pooling to extract the global feature $F_{g}\in {\mathbb{R}}^{512}$ from 32 feature points. According to [9], in order to avoid large differences between facial features, we normalize the features by 2-norm ($L_{2}$):

(9) $$L_{2}\left(F_{g}\right)\to F_{L}\in {\mathbb{R}}^{512}$$

### 3.4. Feature Enhancement Mechanism

In Section 3.3, we obtain normalized facial features $F_{L}\in {\mathbb{R}}^{512}$ (the value of each dimension is between (−1 and 1)). However, not every dimension plays the same role in the recognition task. For example, the larger the eigenvalue of a certain dimension, the higher the recognition contribution of this dimension provides; on the contrary, the smaller the eigenvalue of a certain dimension is, the lower the recognition contribution of this dimension provides. Based on the above phenomenon, we propose a new feature enhancement mechanism to enhance the discrimination of features.

First, take the absolute value of the eigenvalues of each dimension according to Formula (10). Then, use *softmax* to map $\left|F_{L}\right|$ to the probability distribution between (0 and 1). In this step, according to Formula (11), the numerator of eigenvalue with a large absolute value grows fast and the numerator of eigenvalue with a small absolute value grows slowly (because $\left(e^{x}\right)’=e^{x}$). The stretched eigenvalues can improve the discrimination of features. Finally, as shown in Formula (12), we restore the eigenvalues to their original positive and negative distributions.

(10) $$\left|F_{L}\right|=\left(\left|x_{1}\right|,\left|x_{2}\right|,\left|x_{3}\right|,\cdot \cdot \cdot ,\left|x_{512}\right|\right)$$

(11) $$F_{s}=\left(f_{1},f_{2},f_{3},\cdot \cdot \cdot ,f_{i}\right)=softmax\left(\left|F_{L}\right|\right),f_{i}=\frac{e^{\left|x_{i}\right|}}{\sum_{k=1}^{n}e^{\left|x_{k}\right|}}$$

(12) $$F_{S*}=\left(f_{1}^{*},f_{2}^{*},f_{3}^{*},\cdot \cdot \cdot ,f_{512}^{*}\right),f_{i}^{*}=\left\{\begin{array}{c} f_{i},x_{i}\geq 0\\ -f_{i},x_{i}<0 \end{array}\right.$$

We use *softmax* to enhance the eigenvalue in $F_{L}$, but, in order to avoid ignoring some original information in $F_{L}$, we utilize the enhancement parameter λ to linearly add $F_{L}$ and the enhanced feature $F_{s*}$:

(13) $$F_{T}=F_{L}+\lambda F_{S*}$$

**Figure 9:**
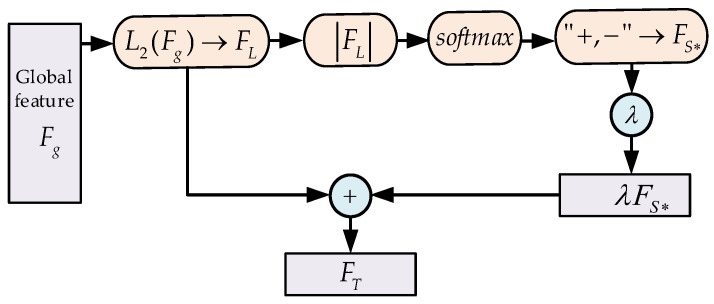
The structure of the feature enhancement mechanism.

In Formula (13), the eigenvalues in $F_{L}$ and $F_{s*}$ are between (−1 and 1), but there is still a large gap. Parameter λ determines the degree of coupling of the two features and also determines the contribution of the proposed feature enhancement mechanism to the $F_{T}$. The structure of feature enhancement mechanism is shown in Figure 9.

### 3.5. Triplet Loss Function

In the feature space, the metric distance between objects is related to the similarity and the training purpose of the face recognition network is to make the same object have a closer metric distance, with a far metric distance between different objects.

In the field of 2D face recognition, FaceNet [9] constructed a triplet loss function and has surpassed humans in accuracy. In this section, we construct a triplet loss based on enhancement parameter λ.

**Figure 10:**
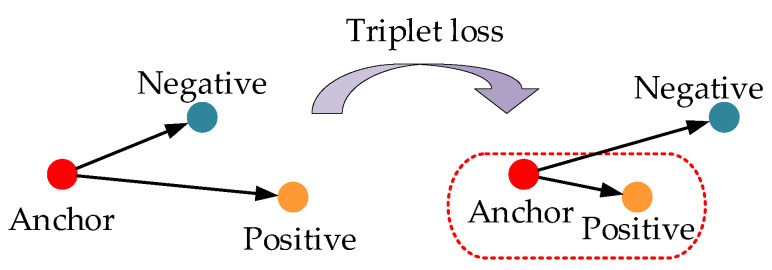
The schematic diagram of triplet loss training process.

The triplet loss function includes three types of samples: anchor samples (Anchor), positive samples (Positive), and negative samples (Negative). The anchor samples and positive samples come from the same object and the negative samples come from different objects. As shown in Figure 10, the purpose of the network is to make the metric distance between the anchor sample ($F^{A}$) and the positive sample ($F^{P}$) with the farthest distance smaller than the anchor sample and the negative sample ($F^{N}$) with the closest distance.

According to Formula (13), face features $F_{T}$ are composed of two parts: $F_{L}$ and $\lambda F_{s*}$. As shown in Formula (12), $F_{L}$ to $\lambda F_{s*}$ is a non-linear change process. If directly using $F_{T}$ for measurement, some original details of the features will be ignored. Therefore, in this section, we construct a new triplet loss according to parameter λ and the training purpose in Figure 10 can be expressed as follows:

(14) $$||F_{L}^{A}-F_{L}^{P}{||}_{2}^{2}+||\lambda F_{S*}^{A}-\lambda F_{S*}^{P}{||}_{2}^{2}+\beta <||F_{L}^{A}-F_{L}^{N}{||}_{2}^{2}+||\lambda F_{S*}^{A}-\lambda F_{S*}^{N}{||}_{2}^{2}$$

where $F_{L}^{A}$, $F_{L}^{P}$, and $F_{L}^{N}$ represent the $F_{L}$ feature (as Formula (9)) of Anchor, Positive, and Negative samples, respectively. $F_{S*}^{A}$, $F_{S*}^{P}$, and $F_{S*}^{N}$ represent the $F_{s*}$ feature (as Formula (12)) of Anchor, Positive, and Negative samples, respectively. λ is the enhancement parameter (as Formula (13)) and β is a margin that is the minimum distance between $||F_{L}^{A}-F_{L}^{P}{||}_{2}^{2}+||\lambda F_{S*}^{A}-\lambda F_{S*}^{P}{||}_{2}^{2}$ and $||F_{L}^{A}-F_{L}^{N}{||}_{2}^{2}+||\lambda F_{S*}^{A}-\lambda F_{S*}^{N}{||}_{2}^{2}$.

In the training process, only samples that do not satisfy Formula (14) are used to optimize the model (the loss of sample that satisfies the Formula (14) is 0):

(15) $$||F_{L}^{A}-F_{L}^{P}{||}_{2}^{2}+||\lambda F_{S*}^{A}-\lambda F_{S*}^{P}{||}_{2}^{2}+\beta >||F_{L}^{A}-F_{L}^{N}{||}_{2}^{2}+||\lambda F_{S*}^{A}-\lambda F_{S*}^{N}{||}_{2}^{2}$$

**Figure 11:**
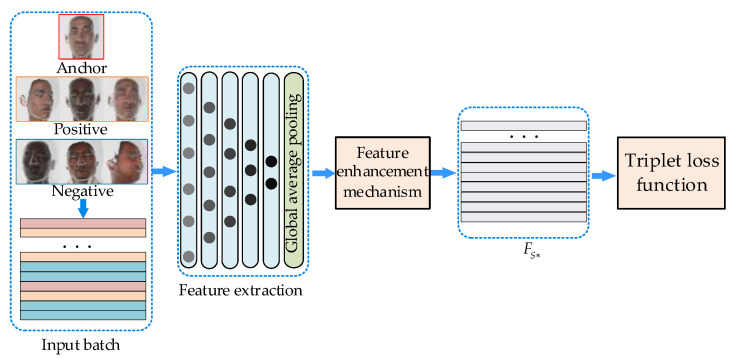
The complete pipeline of our proposed network for face recognition.

The loss function of our model is defined as follows:

(16) $$Loss=\sum^{N}{\left[AP-AN+\beta \right]}_{+}$$

(17) $$AP=||F_{norm}^{A}-F_{norm}^{P}{||}_{2}^{2}+||\lambda F_{en}^{A}-\lambda F_{en}^{P}{||}_{2}^{2}$$

(18) $$AN=||F_{norm}^{A}-F_{norm}^{N}{||}_{2}^{2}+||\lambda F_{en}^{A}-\lambda F_{en}^{N}{||}_{2}^{2}$$

where *N* represents the total number of triplet samples satisfying Formula (15). During the training process, according to the loss function, Anchor and Positive samples with far distance become closer. Anchor and Negative samples with close distance become farther. The whole structure of our network is shown in Figure 11.

Ideally, we want the farthest pair of same objects (hard positive pair) to have a smaller metric distance than the closest different objects (hard negative pair). However, for a large number of training samples, it is difficult to find the hard positive pair and the hard negative pair. Sample selection is very important for the performance of the model. As described in Section 3.3, each point face samples 1024 points as input. According to [8,9], we set the mini-batch in each batch. For a mini-batch, 40 samples are selected from the same subject and we find the hard positive pair in the 40 samples. The hard negative pair is randomly selected from other subjects. The margin β in Formula (14) is computed in each mini-batch. The size of each batch in our network is fixed at 1800. The ADAM optimizer has an initial learning rate of 0.01 for the training of our model.

## 4. Experiments

In this section, we conduct a series of experiments on public datasets to verify the effectiveness of our proposed method. Firstly, we introduce three public datasets CASIA-3D, Lock3Dface, and Bosphoru. Then, we conduct ablation experiments and explore the influence of enhancement parameter λ. Finally, we use our best results for comparison with current advanced methods and analyze the comparison results.

### 4.1. Datasets

CASIA-3D [33]: This dataset used Minolta vivid910 to scan 123 subjects and each subject collected 37 or 38 face images with the influence of different facial expressions, head poses, and light intensities. The dataset has a total of 4626 face samples.

We divide the training set and test set of CASIA-3D according to the method in [26]. Only the frontal face and small pose interference samples are used for experiments, including 1784 samples in the training set and 1783 samples in the test set.

Bosphorus: Savran et al. [34] collected this dataset for studying 2D and 3D face analysis tasks. This dataset, based on the structured light 3D system, collected a total of 4666 samples of facial data from 105 subjects; one-third of the subjects were professional actors and each subject provided 35 types of expressions.

We divide the training set and test set according to the method in [23], in which the training set contains 2403 samples and the test set contains 2263 samples.

Lock3DFace: Zhang et al. [35] collected this dataset by Kinect V2 for 3D face analysis. A total of 5671 samples from 509 subjects were included. According to different scenarios, this dataset is divided into five subsets covering variations in expression (FE), neutral face (NU), occlusion (OC), pose (PS), and time lapse (TM).

We divide the training set and test set of Lock3DFace according to the method in [31], in which the 340 subjects are randomly selected as the training set and the remaining 169 subjects are selected as the test set.

### 4.2. Ablation Experiments

In this section, we first investigate the effectiveness of the proposed feature enhancement mechanism and explore enhancement parameter λ in Formula (13).

In this step, we set λ as a fixed value and explore the effect of λ on the accuracy of the network. The results on CASIA-3D, Bosphorus, and Lock3DFace are reported in Table 1, Table 2 and Table 3.

**Figure 12:**
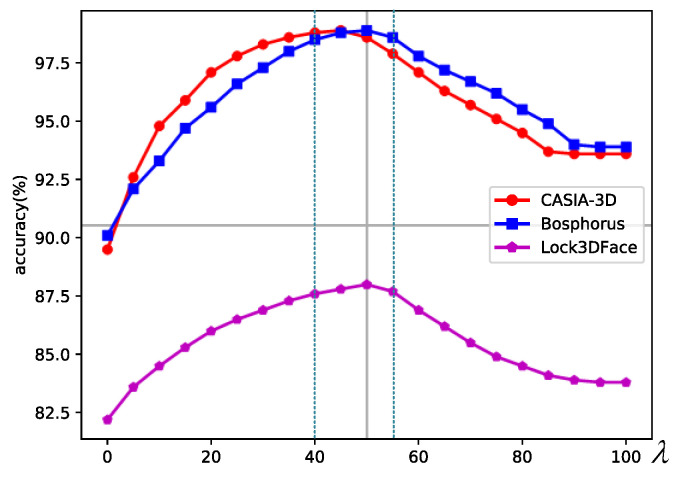
Accuracy change curves with different λ on three datasets.

According to Table 1, Table 2 and Table 3, when λ=50, λ=55, and λ=55 on CASIA-3D, Lock3DFace, and Bosphorus, our network achieves the best accuracy 98.9%, 98.9%, and 88.0%, respectively. Figure 12 intuitively presents the relationship between λ (x-axis) and accuracy (y-axis).

As shown in Figure 12, when λ=0, according to Formula (13), the proposed feature enhancement mechanism is not utilized. With the increase in λ, the feature enhancement mechanism begins to enhance the features and the accuracy of the network begins to increase, which proves that our feature enhancement mechanism can effectively enhance the discrimination of features and improve the recognition accuracy of the network. As λ continues to increase, the accuracy begins to decline. This is because the contribution of $F_{L}$ in Formula (9) becomes small. In this case, features with smaller absolute values will be ignored ($F_{S*}$ is mainly to enhance the features with large absolute value), which will interfere with the accuracy of the network.

Although the best accuracy on the three datasets corresponds to a different λ, according to Figure 12, when $\lambda \in \left[40,55\right]$, the accuracy curves reach a stable peak. In this interval, $F_{L}$ and $F_{S*}$ have the best coupling degree, which can provide the best discrimination for facial features. According to the evaluation method in [9], Table 4 shows the relationship between λ in the peak interval and list the mean accuracy with the standard error of the mean. According to Table 4, the accuracy is relatively stable in this interval for each dataset, which proves that our method has good generalization ability.

As the experimental results show above, we explored the relationship between λ and accuracy and also demonstrated the effectiveness of the feature enhancement mechanism. Then, in the second step, we continue to explore the effectiveness of the distance metric utilized in the triplet loss function. In Section 3.5, instead of taking the $F_{T}$ (in Formula (9)) as a whole, we measure the distance by $F_{L}$ and $F_{S*}$ separately:

(19) $$||F_{T}^{A}-F_{T}^{P}{||}_{2}^{2}\neq ||F_{L}^{A}-F_{L}^{P}{||}_{2}^{2}+||\lambda F_{S*}^{A}-\lambda F_{S*}^{A}{||}_{2}^{2}$$

According to Formula (19), the right part is not equal to the left part. In order to verify the performance of two measurement methods, we conduct a comparison experiment on three datasets; the results are listed in Table 5.

As shown in Table 5, where $L^{*}$ represents the left part of Formula (19) to measure the distance between two features, while L represents the right part. Table 5 lists the mean accuracy with the standard error of the mean on tree datasets. According to Table 5, the results of the two measurement methods are very close but L is higher. This is because the eigenvalue with a smaller absolute value in $F_{T}$ will be ignored and L can be regarded as two kinds of features to measure the distance between two samples, which is better to capture more differences.

### 4.3. Comparison Experiments

The results of ablation experiments prove the effectiveness of our proposed method. In this section, according to [31], we use our best results to conduct comparison experiments with current advanced methods on three public datasets and analyze the results.

**Figure 13:**
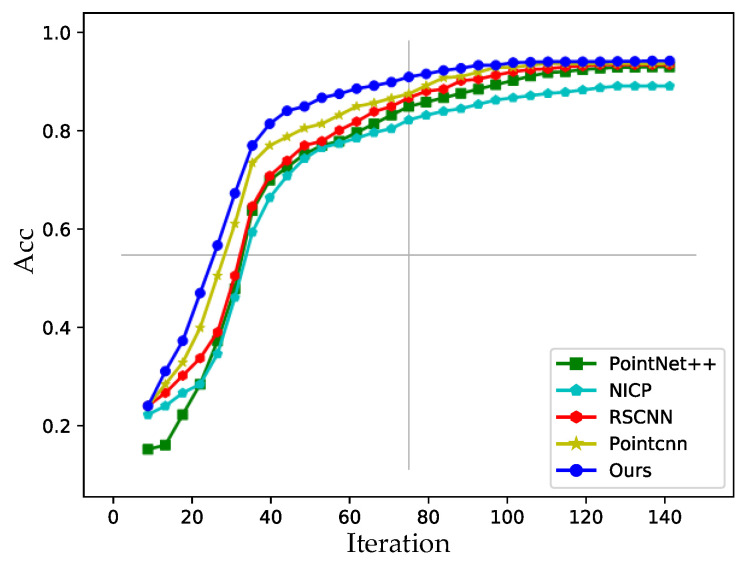
Accuracy change curves during training with different feature extraction network.

Firstly, in order to verify the effectiveness of the proposed ψ−conv network, we use different point clouds based networks to extract facial features and perform face recognition under the same setting on CASIA-3D. The accuracy curves in the training process are shown in Figure 13 and the results are listed in Table 6.

As shown in Figure 13, during the training process, our accuracy curve is higher than other methods and, as the results listed in Table 6, our method also achieves the best accuracy on the test set, which prove the effectiveness of our ψ−conv network. Compared with the method in [8], our network has a similar architecture but adds a local feature descriptor. The comparison results with the method in [8] prove that our network based on a local feature descriptor can better obtain facial fine-grained features and is more conducive to improving the accuracy of the model.

Table 7, Table 8 and Table 9 list the comparison results with the latest face recognition methods on three datasets, respectively.

The results in Table 7 and Table 8 show that, under different datasets, our accuracy is higher than other methods.

**Figure 14:**
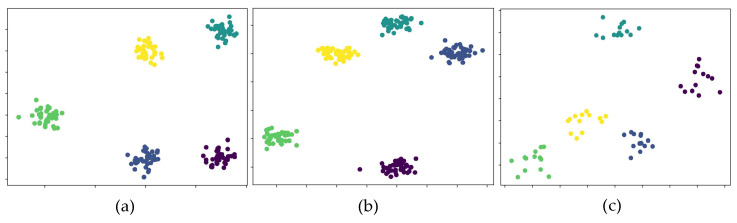
T-SN examples of face recognition, where (**a**–**c**) represent the classification results on CASIA-3D, Bosphorus, and Lock3DFace, respectively.

As described in Section 4.1, Lock3DFace has five subsets: expression changes (FE), normal face (NU), partial occlusion (OC), head pose changes (PS), and time lapse (TL). In order to intuitively verify the performance of our method in different scenarios, we conduct a comparison experiment on the first four subsets: FE, NU, OC, and PS. The results are shown in Table 9. According to the results, in the NU subset, which has no other interference, Jiang et al. [31] achieves the best accuracy, but in the OC and PS subsets, our method achieves the best accuracy, which proves that our network is better to cope with partial occlusions and head pose interference. Figure 14 shows the t-SN example of our network for face recognition on three datasets (each dataset selects five subjects for classification and each color represents one subject). As shown in Figure 14, the classification results on CASIA-3D and Bosphorus are more convergent, but on Lock3DFace are more discrete. This is because there are fewer samples for each subject in Lock3DFace and there is also more interference for samples. However, according to Figure 14c, our method can still distinguish different objects clearly on Lock3DFace.

Apart from the accuracy, the time cost is also an important indicator for measuring the efficiency of the network. Table 10 lists the comparison results of different methods in terms of time costs. In Table 10, “Ours *” represents our method without the feature enhancement mechanism. Compared with “Ours *”, our time cost is very close. This is because the feature enhancement adopts the *softmax* function to stretch the features, the calculation complexity is low, and no additional network parameters are added. The comparison results in Table 10 show that our network also maintains good real-time performance.

## 5. Conclusions

Since point clouds lack detailed textures and since face recognition require fine-grained representation of features, this paper proposes a new operator, ψ−conv, based on the local feature descriptor to realize fine-grained feature extraction of disordered point clouds by a convolutional neural network and constructs the feature enhancement mechanism to improve feature discrimination; meanwhile, the triplet loss function is adopted to optimize the network. In order to verify the performance of our method, we conducted experiments on the CASIA-3D, Lock3Dface, and Bosphorus datasets. The results of the ablation experiments prove that the feature enhancement mechanism and the triplet loss can effectively improve the recognition accuracy of the model. The results of the comparison experiments show that our network outperforms current advanced methods and can better cope with the interference of face expressions, partial occlusions, and head pose changes. Meanwhile, our network also has good real-time performance and can be applied in real scenarios. However, when the pose interference is too large due to the lack of some facial features, the accuracy of our method is still insufficient. We will further explore new methods to improve the accuracy under large pose interference and investigate new algorithms for 3D face analysis, such as head pose estimation, expression recognition, face detection, and other 3D visual tasks in real applications.

## Figures and Tables

**Table 1:** Performance evaluation with different λ on CASIA-3D.

λ	0	10	15	20	25	30	35	40	45	50
Acc (%)	89.5	94.8	95.9	97.1	97.8	98.3	98.6	98.7	98.8	98.9
λ	55	60	65	70	75	80	85	90	95	100
Acc (%)	98.6	97.9	97.1	96.3	95.7	95.1	94.5	93.7	93.6	93.6

**Table 2:** Performance evaluation with different λ on Bosphorus.

λ	0	10	15	20	25	30	35	40	45	50
Acc (%)	90.1	93.3	94.7	95.6	96.6	97.3	98.0	98.3	98.5	98.8
λ	55	60	65	70	75	80	85	90	95	100
Acc (%)	98.9	98.6	97.8	97.2	96.7	96.2	95.5	94.9	94.0	93.9

**Table 3:** Performance evaluation with different λ on Lock3DFace.

λ	0	10	15	20	25	30	35	40	45	50
Acc (%)	82.2	84.5	85.3	86.0	86.5	86.9	87.3	87.5	87.6	87.8
λ	55	60	65	70	75	80	85	90	95	100
Acc (%)	88.0	87.7	86.9	86.2	85.5	84.9	84.5	84.1	83.9	83.8

**Table 4:** Accuracy of the different λ on three datasets.

λ	40	45	50	55
CASIA-3D (Acc%)	98.66±0.04	98.77±0.03	98.89±0.01	98.55±0.05
Bosphorus (Acc%)	98.23±0.07	98.47±0.03	98.78±0.02	98.88±0.02
Lock3DFace (Acc%)	87.38±0.12	87.48±0.12	87.70±0.10	87.91±0.09

**Table 5:** Accuracy of the different metric distance on CASIA-3D.

Dataset	L* Acc (%)	*L* Acc (%)
CASIA-3D	98.27±0.03	98.89±0.01
Bosphorus	98.08±0.02	98.88±0.02
Lock3DFace	87.39±0.11	87.91±0.09

**Table 6:** Comparison of accuracy achieved by different feature extraction network on the test set of CASIA-3D.

Methods	Acc (%)
PointNet++ [6]	95.6
NICP [36]	90.3
RSCNN [37]	95.9
Pointcnn [8]	97.5
Ours	98.9

**Table 7:** Comparison of accuracy achieved by different methods on CASIA-3D.

Methods	Acc (%)
Chouchane et al. [18]	96.8
Dutta et al. [23]	98.2
Gao et al. [26]	97.6
Cao et al. [28]	97.9
Ours	98.9

**Table 8:** Comparison of accuracy achieved by different methods on Bosphorus.

Methods	Acc (%)
Zhang et al. [17]	93.0
Soltanpour et al. [21]	97.3
Dutta et al. [23]	98.5
Cao et al. [28]	98.0
Ours	98.9

**Table 9:** Comparison of accuracy achieved by different methods on Lock3DFace.

Methods	FE (%)	NU (%)	OC (%)	PS (%)	Total (%)
K. He et al. [20]	96.1	99.3	54.9	61.4	76.6
C.S et al. [19]	93.6	99.0	57.0	54.1	74.4
M.S et al. [38]	95.7	98.9	61.4	69.9	79.5
Mu et al. [22]	98.1	99.6	78.1	70.4	84.2
Jiang et al. [31]	98.5	99.5	80.1	73.7	87.2
Ours	98.5	99.3	82.6	74.9	88.0

**Table 10:** Comparison of time costs, where “Ours *” represents our method without the feature enhancement mechanism.

Methods	fps
Dutta et al. [23]	7
M.S et al. [38]	6
Xiao et al. [1]	117
Xiao et al. [32]	125
Mu et al. [22]	136
Jiang et al. [31]	118
Ours *	139
Ours	125

## Data Availability

Not applicable.
